# Therapeutic Itineraries of the Homeless Population for the Diagnosis and Treatment of COVID-19

**DOI:** 10.1590/0034-7167-2025-0349

**Published:** 2026-11-06

**Authors:** Olívia Paulino Pinto, Ana Patrícia Pereira Morais, Mardenia Gomes Vasconcelos Pitombeira, Ana Paula Cavalcante Ramalho Brilhante, Maria Rocineide Ferreira da Silva

**Affiliations:** I Universidade Estadual do Ceará. Fortaleza, Ceará, Brazil; II Centro Universitário Christus. Fortaleza, Ceará, Brazil

**Keywords:** Access to Health Care, COVID-19, Health Policy, Ill-Housed Persons, Health Evaluation., Ruta Terapéutica, COVID-19, Política de Salud, Personas con Mala Vivienda, Evaluación en Salud.

## Abstract

**Objective:**

to understand the therapeutic itineraries of homeless people in their search for the diagnosis and treatment of COVID-19.

**Methods:**

a qualitative and retrospective study was conducted in Fortaleza, Ceará, Brazil, through semi-structured interviews. Data were organized using IRAMUTEQ^®^ and analyzed through the “therapeutic itineraries” analytical framework, guided by Leny Trad’s holistic analysis of therapeutic itineraries and Kleinman’s healthcare system model.

**Results:**

the discovery of the diagnosis was guided by identification in formal healthcare settings and informal support networks. Primary Health Care was not accessed, indicating that this level of care played a less active role and prioritized a more biomedical approach to care. Participants reported the use of alcohol and other drugs to relieve COVID-19 symptoms.

**Final considerations:**

the therapeutic itineraries revealed deficiencies in participants’ access to healthcare services, as well as shortcomings in service resolution and comprehensiveness across the network.

## RESUMEN

**Objetivo:**

comprender los itinerarios terapéuticos de las personas sin hogar que buscan diagnóstico y tratamiento para la COVID-19.

**Métodos:**

se realizó un estudio cualitativo y retrospectivo en Fortaleza, Ceará, mediante entrevistas semiestructuradas. Los datos se organizaron en IRAMUTEQ^®^ y se analizaron utilizando la matriz de análisis de “itinerarios terapêuticos”, guiada por el análisis holístico de itinerarios terapéuticos de Leny Trad y el sistema de atención de salud de Kleinman.

**Resultados:**

el descubrimiento del diagnóstico se guió por el descubrimiento en espacios de salud formales y redes de apoyo informales. No se accedió a la atención primaria de salud, lo que indica que este nivel de atención fue menos frecuente, priorizando un enfoque más biomédico. Los participantes informaron haber consumido alcohol y otras drogas para aliviar los síntomas de la COVID-19.

**Consideraciones finales:**

los itinerarios evidenciaron deficiencias en el acceso, la resolución y la integralidad de los servicios para los participantes.

## INTRODUCTION

Homelessness is characterized as a global human rights crisis, marked by the denial of social identity, lack of housing, criminalization, and deprivation of socioeconomic rights. The National Policy for the Homeless Population defines homeless persons (HPs) as a heterogeneous population group that shares common experiences of extreme poverty, broken or fragile family ties, and the use of public spaces as places of residence and/or livelihood^(1-3)^.

HPs are subject to social and programmatic vulnerability, with persistent and recognized difficulties in accessing the Brazilian Unified Health System (SUS - *Sistema Único de Saúde*) and social support services. This results in high susceptibility to infections due to the precarious conditions encountered on the streets^(4)^. In this context, the emergence of COVID-19 (Coronavirus Disease 2019) intensified HPs’ vulnerability, as the infection caused by this virus proved to be highly contagious and lethal, particularly among susceptible individuals^(4,5)^.

Therefore, although the disease can affect any individual, macro-social issues are related to its worsening, making it undeniable that the most affected groups are those experiencing vulnerability^(6)^. Socioeconomic vulnerability increased significantly after the pandemic, contributing to the growth of the homeless population^(7)^, reaching approximately 221,000 individuals, which already represented a 140% increase since September 2012^(8)^. Regarding COVID-19, Brazil had recorded 39,318,227 cases and 716,626 deaths by 2026. These figures demonstrate the magnitude of the pandemic since its onset in 2020 and its profound impact on the health of Brazilians^(9,10)^.

This context raises important reflections on the care provided to HPs during the pandemic. To this end, this investigation adopted the theory and practice of therapeutic itineraries (TIs), which have been used to understand how specific social groups navigate their pathways toward obtaining the care they need^(11)^. This study is relevant because it may contribute to improving care pathways across different healthcare networks, ensuring social rights and allowing users to express their trajectories within the healthcare system. It also contributes to the production of knowledge in public health, particularly in the fields of social sciences, planning, management, and public policy assessment.

## OBJECTIVE

To understand the TIs of HPs in their search for the diagnosis and treatment of COVID-19.

## METHOD

### Ethical aspects

The study complied with the regulations established by Resolution 466/2012 of the Brazilian National Health Council, which governs research involving human subjects, and Resolution 510/2016, which establishes specific guidelines for the human and social sciences. The study was approved by the Research Ethics Committee of *Universidade Estadual do Ceará*. To access participants, authorization was obtained from the Municipal Department of Human Rights and Social Development to conduct the study at the Specialized Reference Center for the Homeless Population (*Centro Pop*), and consent for conducting interviews at *Casa da Sopa* was granted by the responsible volunteers. During the approach to HPs, the Informed Consent Form (ICF) was presented. To ensure participants’ confidentiality and anonymity, numerical codes were used in the data collection instruments.

### Theoretical-methodological framework

The analytical framework adopted in this study was that of TIs. Among the theoretical references on TIs, the holistic analysis of TIs proposed by Leny Trad^(12)^ was selected to interpret the material. This model presents four interdependent analytical dimensions: symbolic dimension - responsible for meanings related to health, illness, and care; contextual dimension - representing living conditions, economic, social, and cultural capital, as well as social support networks; experience assessment - encompassing organizational, relational, effectiveness, and therapeutic aspects; therapeutic rationality and practices - related to domestic practices and other therapeutic care settings^(12)^. In addition, Kleinman’s explanatory models^(13)^ were considered in the analysis. These models are defined as conceptions of illness and treatment approaches employed by all those engaged in a health-illness process.

### Study design

This was a qualitative study with a retrospective approach. The study complied with the items recommended by the Standards for Reporting Qualitative Research.

### Study setting

The study was conducted in Fortaleza, Ceará, Brazil, a city located in Northeastern Brazil that, according to the 2025 census, has 2.57 million inhabitants and approximately 10,037 HPs. The study was carried out in two settings: *Centro Pop* and *Grupo Espírita Casa da Sopa. Centro Pop* provides access to official documentation, the Unified Registry for Social Programs (*Cadastro Único para Programas Sociais*), personal hygiene services, socioeducational and artistic activities, as well as specialized assistance, including healthcare services. *Casa da Sopa* is a reference institution classified as a Civil Society Organization that works in the development of social care, promoting the defense of HPs’ rights and offering activities in the field of social assistance^(14)^.

### Data source

The study included individuals who had experienced homelessness during the 2020-2023 pandemic period, had received a diagnosis of COVID-19, did not need to provide proof of testing, and were older than 18 years of age. Individuals were excluded if they were unable to communicate due to cognitive disorders that made communication during data collection impossible, if they did not speak Portuguese, or if they were under the influence of alcohol or other psychoactive substances. Participants were selected using the snowball sampling technique, a non-probabilistic sampling method based on chain-referral recruitment^(15)^. The criterion used to conclude participant recruitment was theoretical saturation of the testimonies. No approached participant refused to participate.

### Data collection and organization

The interviews were conducted using a semi-structured script between September and October 2023 and between January and March 2024. They were segmented into two parts: the first refers to sociodemographic data; and the second refers to the script, which contains the following trigger questions: can you tell me about your search to discover and learn about your illness? Can you tell me about the path you took to receive treatment? Why did you choose this path? These interviews were conducted by the principal researcher during her master’s degree, individually and according to the participant’s availability. They lasted between 30 and 40 minutes, were recorded after verbal consent and signing of the ICF, and were transcribed in full by the researcher. Data were organized using *Interface de R pour les Analyses Multidimensionnelles de Textes et de Questionnaires* (IRAMUTEQ^®^), a free software package. The tool enabled identification of the semantic roots of words and extraction of textual classes, allowing the consolidation of the Descending Hierarchical Classification (DHC).

### Data analysis

The analysis of the presented pathways was transcribed and initially identified by order of participant approach. After this stage, the material was read in its entirety and organized using the chosen tool. This reading was necessary for the constitution of the *corpus*, formulation, and reformulation of assumptions. The theoretical foundation was provided by the TIs framework. Through TIs, it is possible to understand that individuals base their choices on their needs, beliefs, life perspectives, educational level, and stigmas; in other words, they design and define their own pathways within the healthcare system, regardless of what has been previously established^(16)^.

The narratives of the six participants were compiled into a *corpus* and processed using IRAMUTEQ^®^ software. DHC identified six textual classes, 109 textual segments, 3,701 occurrences, and 960 active forms, with an average presentation of ≥ 3:147. It is noteworthy that the text *corpus* had an 80.73% utilization rate, with vocabulary showing statistically significant association (p<0.0001). Four categories were generated: Narrating the onset of the disease and the feelings involved in its discovery; Social support network for homeless people during the pandemic; Alcohol and other drug use among homeless people during the pandemic; Considerations on Primary Health Care in the care of homeless people with COVID-19.

## RESULTS

Six individuals were interviewed, one at *Centro Pop* and five at *Grupo Espírita Casa da Sopa*. The participants and the TIs they followed are described below: P1 was a 57-year-old man, single, Catholic, with one child, born in Fortaleza, self-identified as White, cisgender, heterosexual, and had completed high school education. He had been experiencing homelessness for 22 years and was receiving Social Rent assistance. COVID-19 was diagnosed in 2021 through systematic testing conducted by the Catholic Community Shalom during food distribution in a public square. Before receiving a positive diagnosis, the participant had undergone ten tests, all with negative results. After learning of the diagnosis, his first action was to buy alcoholic beverages and adhere to isolation measures. In his account, the participant stated that he was asymptomatic, although he was unsure because of his alcohol consumption. He believed that alcohol could have harmful effects against the coronavirus. During this period, he did not seek healthcare services and, after remaining isolated for two weeks, returned to the public space he had previously occupied (Figure 1).

**Figure 1:**
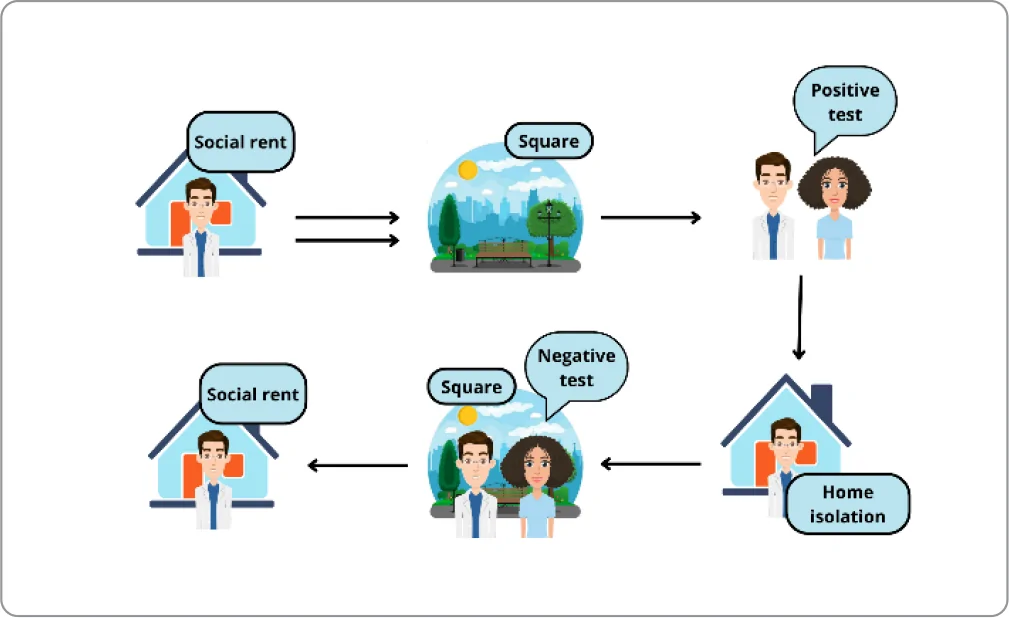
P1’s therapeutic itinerary. Fortaleza, Ceará, Brazil, 2026

P2 was a 35-year-old man, Catholic, divorced, with three children, born in Fortaleza, self-identified as mixed-race, cisgender, heterosexual, had not completed elementary school, and worked as a recyclable materials collector. He had been experiencing homelessness for more than four years and was receiving Social Rent assistance. The participant attributed the origin of the infection to a previous hospital admission, reporting that he discovered the disease shortly after discharge. The suspicion of COVID-19 arose after the onset of respiratory discomfort and loss of smell and taste. In response to these symptoms, he went to a 24-hour Emergency Care Unit (ECU), underwent testing, and was advised by healthcare professionals to remain in isolation. The participant believed that drug use could contribute to his recovery. Thus, upon returning home, he used cocaine to relieve respiratory discomfort. According to his account, there was initial improvement followed by worsening symptoms. When the respiratory discomfort intensified, he returned to the ECU, but healthcare professionals maintained the same intervention. Afterward, he returned to the unit three more times. He reported that God and cigarettes helped him endure that period (Figure 2).

**Figure 2:**
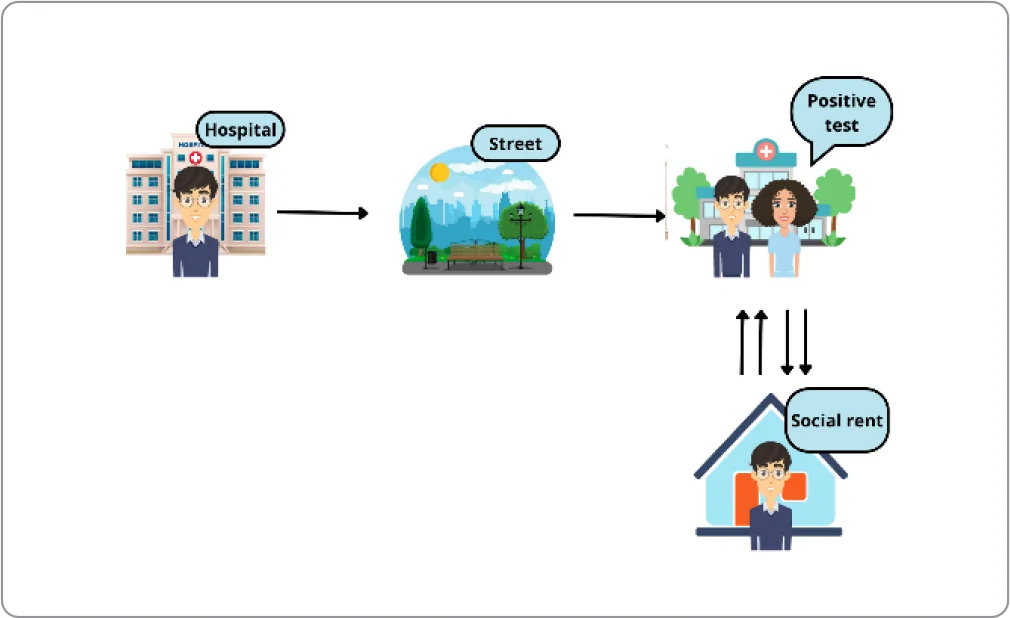
P2’s therapeutic itinerary. Fortaleza, Ceará, Brazil, 2026

P3 was a 39-year-old man, Evangelical Christian, single, with no children, born in Fortaleza, self-identified as mixed-race, cisgender, heterosexual, and had not completed elementary school. He experienced homelessness for seven months during the pandemic and had been in this condition for one year. COVID-19 infection occurred in 2020 and manifested with fever and respiratory discomfort. At that time, he was experiencing homelessness and was also deprived of liberty. The diagnosis was confirmed through a Reverse Transcription Polymerase Chain Reaction test at the time of admission to the prison system. After diagnosis, he was referred to a tertiary healthcare facility and subsequently transferred to another hospital, where he underwent isolation and treatment. Upon completion of treatment, he returned to the original facility. During this period, he reported that his primary source of support was the nursing team (Figure 3).

**Figure 3:**
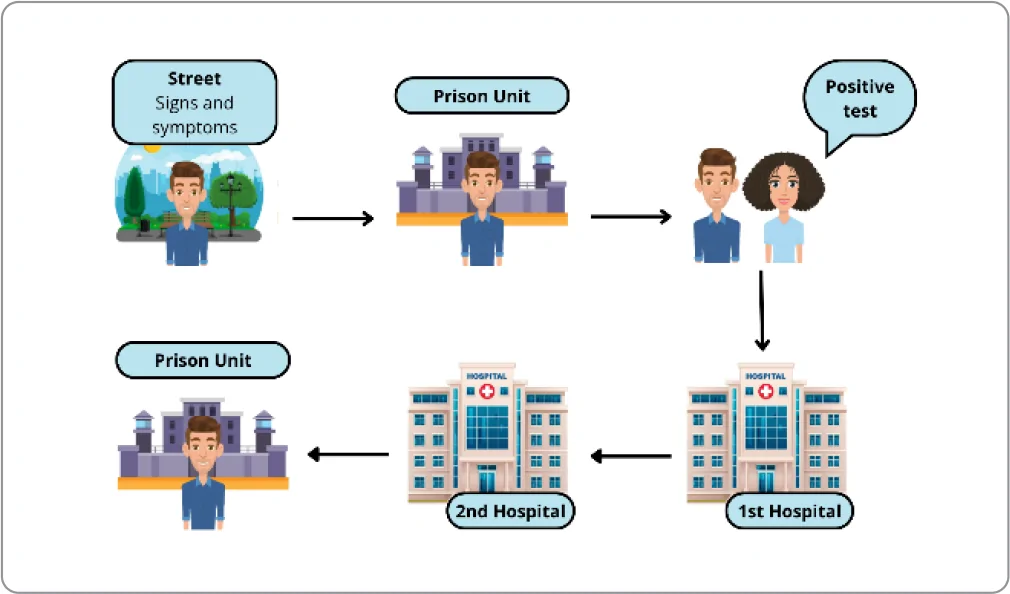
P3’s therapeutic itinerary. Fortaleza, Ceará, Brazil, 2026

P4 was a 38-year-old man, Evangelical Christian, living in a stable union, with three children, born in Fortaleza, self-identified as mixed-race, cisgender, heterosexual, had completed high school education, and was unemployed. He had been experiencing homelessness for ten years. His TI began when he developed fever and body aches. Upon noticing the symptoms, he sought help from his sister, who accompanied him to a 24-hour ECU. The participant reported having a special bond with this facility, frequently seeking care there and being recognized by healthcare professionals. At the ECU, the diagnosis of COVID-19 was confirmed, and hospital admission was recommended. He was transferred to a tertiary healthcare facility and, during this period, experienced respiratory discomfort and fever. After 20 days, he was discharged and returned to the streets (Figure 4).

**Figure 4:**
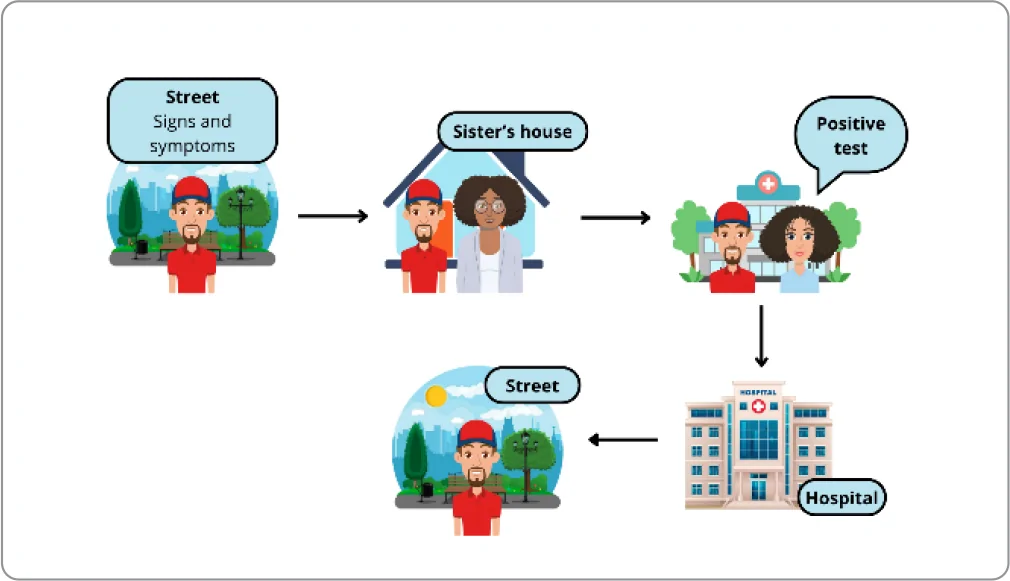
P4’s therapeutic itinerary. Fortaleza, Ceará, Brazil, 2026

P5 was a 27-year-old man with no religious affiliation, married, with four children, born in Fortaleza, self-identified as mixed-race, transgender, and bisexual. He had been experiencing homelessness for nine years and, at the time, was a beneficiary of Social Rent assistance. COVID-19 infection occurred in 2020. He was living on the streets when he developed fever and respiratory discomfort and, although he suspected COVID-19, did not seek healthcare services. The diagnosis was confirmed through testing after admission to the prison system. Following diagnosis, he was placed in isolation and described the experience as precarious, including exposure to tear gas and water being sprayed inside the cell by correctional officers. The food provided was spoiled. During this period, he reported receiving no support and, after completing the isolation period, returned to the original facility. He stated that his support came from God (Figure 5).

**Figure 5:**
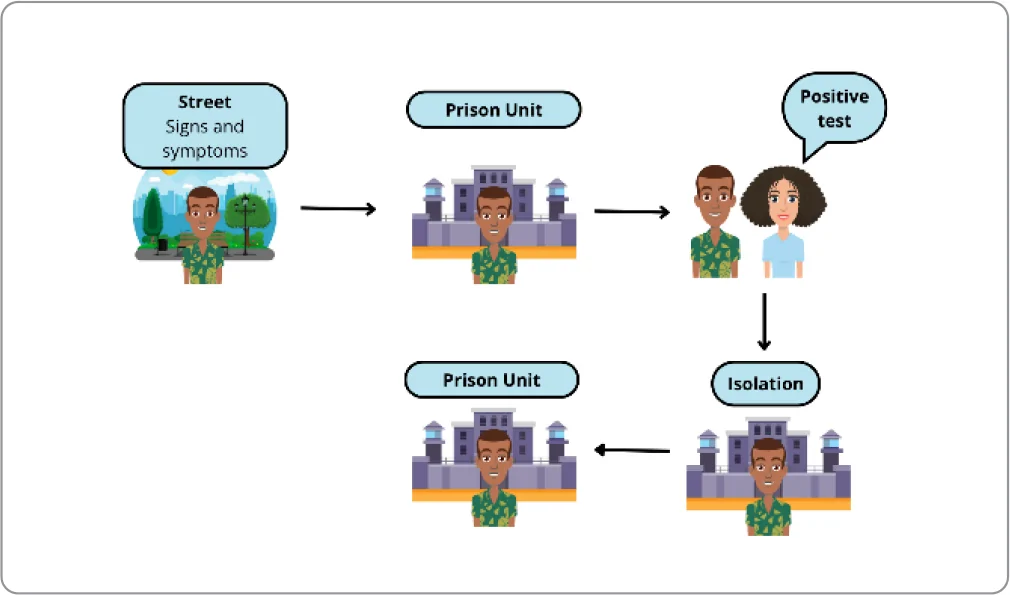
P5’s therapeutic itinerary. Fortaleza, Ceará, Brazil, 2026

P6 was a 54-year-old man, Evangelical Christian, widowed, with no children, born in Fortaleza, self-identified as Black, cisgender, heterosexual, and illiterate. He had been experiencing homelessness for six years. He was assaulted on the street and sustained a leg fracture caused by a blunt object, requiring surgical intervention. Shortly after the procedure, he developed symptoms including respiratory discomfort, dizziness, and malaise. Following a positive diagnosis, he was transferred to an isolation area and, in addition to the disease symptoms, reported hearing voices and seeing shadowy figures hidden in the room. According to the participant, his greatest source of support was his pastor, who assisted him after hospital discharge. He completed treatment at the Recovery Home of the Seventh-day Baptist Church, a facility that helped him cope with post-COVID symptoms and recover from his injury. In his testimony, he stated: “I felt despair and wanted to kill myself [...] actually, I always kneel down, you know? I ask God for a lot of strength” (Figure 6).

**Figure 6:**
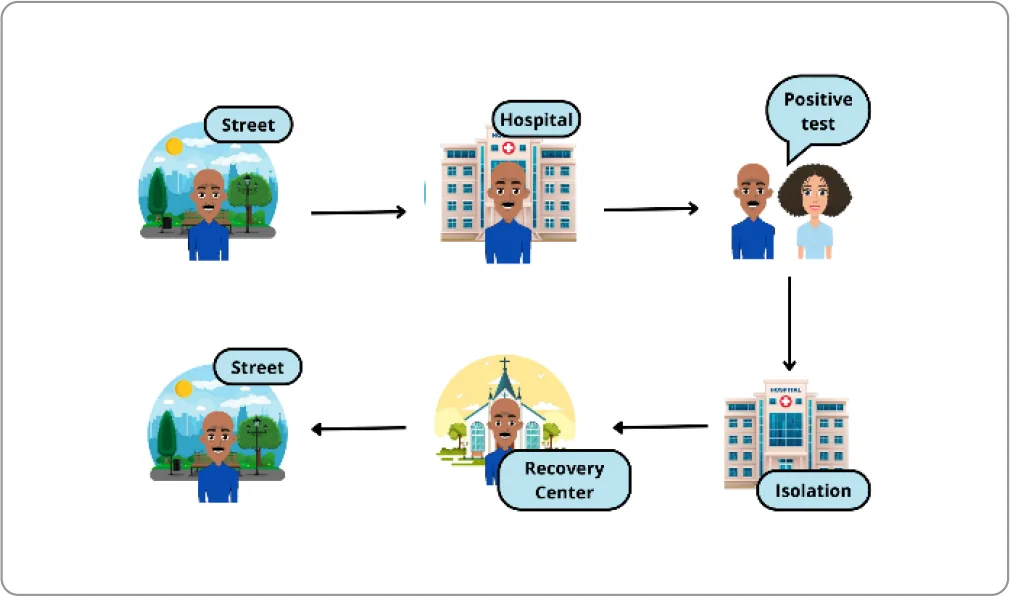
P6’s therapeutic itinerary. Fortaleza, Ceará, Brazil, 2026

## DISCUSSION

### Narrating the onset of the disease and the feelings involved in its discovery

The TIs experienced by the participants in this study were not guided solely by their search for care, but also by the discovery of the diagnosis in formal healthcare settings or through informal support networks. Therefore, the importance of access to the formal healthcare network for this population should be emphasized, since individuals who were not reached by healthcare interventions remained vulnerable to worsening health conditions and disease transmission.

Participants’ narratives revealed that respiratory discomfort became, from their perspective, an identifying symptom of the disease. However, most individuals present mild (40%) or moderate (40%) symptoms, and only 15% develop symptoms requiring oxygen support^(17)^. Thus, the search for healthcare among those with mild cases may have been hindered by a lack of knowledge regarding signs and symptoms. Limited knowledge about signs and symptoms, delays in accessing healthcare services, the absence of referral services, and structural and operational failures within the Primary Care Network make access to diagnosis more difficult^(18)^.

During the pandemic, several factors contributed to the low positivity rates identified among this population. Difficulties in access may have resulted in undiagnosed cases, while other factors, such as the lack of identification documents and the absence of specific markers, also affected the recording of deaths among HPs in census data. There were no records of the number of COVID-19 cases and deaths among these individuals in Fortaleza, which hindered Epidemiological Surveillance actions^(19)^.

The reflections generated through the construction of TIs reveal gaps in care and shortcomings in the operationalization of the healthcare system. Therefore, it must operate beyond a functionalist perspective, taking social demands into account and adapting the provision of healthcare practices and services accordingly^(16)^.

Users’ feelings and perceptions played an important role in recovery and in their behavior when facing illness. As illustrated by participant 6, the disease was perceived as frightening, yet also through a perspective of hope and overcoming adversity. Given the widespread panic partly disseminated by the media, negative feelings were expected. However, most participants demonstrated positive coping strategies, generally motivated by religious beliefs. Such beliefs primarily serve to confront threats and renew strength in the struggle for survival^(20)^.

### Social support network for homeless people during the pandemic

A social support network is defined as the set of individuals with whom meaningful relationships are established and from whom support is perceived and/or received. This concept is extremely important for understanding the production of healthcare because, when a network is integrated and strong, it has the capacity to foster resilience in times of crisis^(21)^. To understand the therapeutic choices made by individuals, it is necessary to identify these networks. They may consist of close personal contacts as well as formal and informal care settings, since it is through these networks that individuals experience illness and mobilize resources for care^(22)^.

The contextual dimension was analyzed here, as it encompasses information related to individuals’ living environments, considering local social, healthcare, and infrastructural conditions. It is important to assess the extent and characteristics of available care resources, taking into account both formal and informal service modalities^(12)^. The pandemic required adaptation and reorganization of healthcare delivery at all levels of complexity to ensure effective care for individuals diagnosed with COVID-19. Measures included expanding hospital bed capacity, hiring healthcare professionals, providing personal protective equipment, creating isolation facilities, developing new care pathways and protocols, offering training programs, and implementing other necessary actions^(23)^.

To this end, a synthesis was produced of the formal and informal support networks identified in the participants’ accounts regarding the resources used for diagnosis and treatment. Among those mentioned, the professional sector was the most prominent in the search for healthcare. Facilities such as 24-hour ECUs and hospitals were reported as the most frequently accessed services.

The influence of the folk and informal sectors was also observed in providing support during illness, particularly through friends, family members, and religious organizations. Social actors sought care and treatment through multiple articulations within their social networks, creating unique TIs characterized by uncertainty, hesitation, and the use of multiple resources. Finally, these sectors should not be viewed separately. Kleinman emphasizes the need for integration among sectors to ensure comprehensive care^(13)^.

### Considerations on Primary Health Care in the care of homeless people with COVID-19

In mapping the resources that compose the professional sector, most participants reported accessing secondaryand tertiary-level healthcare services, as well as nongovernmental institutions. However, while these actors contributed to the realization of HPs’ rights, the findings also reveal gaps left by Primary Health Care (PHC) and referral services^(24)^. The potential of PHC in responding to crises is decisive, particularly through community orientation and the recognition of needs within territories, contributing through epidemiological analysis and playing an essential role in protecting vulnerable populations such as HPs^(24)^.

Nevertheless, the documents published by the Ministry of Health (MoH) emphasized the clinical management of COVID-19, establishing rigid testing protocols restricted to symptomatic cases and classifying suspected cases as nonspecific influenza-like illness. Consequently, PHC did not receive sufficient structure and/or guidance from MoH documents to establish active case-finding strategies or coordinate testing efforts, leading to the concentration of testing within hospital settings^(24)^. Regarding the Fortaleza contingency plan, the document highlights the care and regular follow-up of patients with suspected or confirmed COVID-19. In other words, the focus was placed primarily on a biomedical dimension of care^(25)^.

Directing attention to the study population, in 2020 the Ceará State Health Department issued a technical note containing recommendations for HPs. The purpose was to guide prevention and protection measures for this population across different levels of care. In municipalities lacking Street Outreach Clinics, a PHC service designed to provide comprehensive care to HPs, PHC would be responsible for offering specialized care to this population^(26)^. However, participants’ narratives revealed access to other levels of care, suggesting that the recommended measures had little impact or were not effectively implemented within the municipality^(27)^.

During health crises, PHC has the capacity to assume a leading role due to its connection with the territory, its effectiveness in responding to epidemics, and its commitment to comprehensive care. These characteristics make it particularly important in addressing collective illness caused by an unknown disease, especially in contexts aggravated by social vulnerability, food insecurity, unemployment, and violence. Nevertheless, a weakening of PHC actions in responding to the crisis was observed, resulting in the imposition of a “non-place” for this level of care despite its significant potential within the healthcare network^(16)^.

Countries in Latin America, including Brazil, were already facing economic, political, and social challenges. Public management and the timeliness of responses were fundamental in guiding the course of the pandemic. Even countries with substantial economic resources experienced illness and deaths among their populations as consequences of decisions made by local and health authorities^(28)^.

### Alcohol and other drug use among homeless people during the pandemic

Homelessness is associated with health, social, and legal vulnerabilities, which are exacerbated by the misuse of psychoactive substances. Within this context, substance use often serves the purpose of facilitating socialization and alleviating hunger and cold^(29)^. This contextual dimension, described within the analytical framework, encompasses inequalities, barriers to accessing basic services, and insufficient public policies, all of which hinder efforts to overcome vulnerability^(12)^.

Substance use among this population carries multiple meanings and symbols, highlighting the need for a deeper understanding so that public policies can be developed in a contextualized and effective manner^(30)^.

In some cases, substance use is perceived by individuals themselves as therapeutic, serving as a strategy to relieve or cure pain and psychological suffering. This reflects the symbolic dimension, which encompasses representations and meanings related to the health-disease process. In this context, positive perceptions of drug use directly affect health and the illness experience of these individuals. From another analytical perspective, the therapeutic pathway involves examining the underlying rationalities and therapeutic practices present in different care settings. Field research revealed deficits in self-care and social support, conditions reflected in the use of psychoactive substances as a therapeutic response to the disease^(12)^.

It is necessary to address substance use within the TIs described because social isolation and the new dynamics experienced during the pandemic created a favorable environment for substance use, particularly among individuals who had previously consumed alcohol and other drugs^(31)^. The vulnerability generated by isolation and uncertainty about the disease corroborates the findings of Jr. and Ximenes^(32)^, who demonstrated that drug use contributes to mediating suffering and discomfort associated with illness by providing immediate pleasure, relief, and a sense of familiarity, particularly in cases of prolonged psychoactive substance use.

The meanings attributed by participants reflect a diversity of contexts, symbolisms, and rationalities. However, these TIs also highlight gaps left by different levels of healthcare. Compared with the contexts of North America and Europe, Brazil experienced less support and urgency in allocating resources. The expansion of harm reduction and treatment services, together with intersectoral collaboration among services, enabled increased access to mental healthcare services in those settings^(33)^.

### Study limitations

The main limitation of this study was the challenge of approaching the target population, as most participants were unaware of their own diagnosis. A more comprehensive assessment of the topic would require investigations in other regions and a larger sample size.

### Contributions to nursing, health, or public policies

In the fields of public health and nursing, the use of TIs enables a more accurate analysis of the needs of HPs because it is through the users’ perspectives that contingency plans capable of addressing health demands in emergency situations can be developed. Within this framework, TIs constitute an assessment method that enables the shared construction of public policies.

## FINAL CONSIDERATIONS

The reconstruction of these pathways revealed that the search for diagnosis was shaped by the severity of illness and by relationships established before the pandemic, whether with healthcare facilities, family members, or religious figures. The findings highlighted the lack of structure, organization, and coordinated networks to support HPs’ access to healthcare, demonstrating weaknesses in the effectiveness and comprehensiveness of services. Considering the trajectories described by participants, the fragmentation of the healthcare network became evident, leaving treatment concentrated within secondaryand tertiary-level services as well as nongovernmental institutions.

It is important to strengthen collaboration among healthcare networks to ensure more effective responses during times of crisis. The construction of TIs has the potential to foster, strengthen, and ultimately improve initiatives aimed at articulation and cooperation among management spaces, healthcare providers, services, and diverse care settings.

## Data Availability

The research data are available upon request.
