# Supplementary material for: Therapeutic Itineraries of the Homeless Population for the Diagnosis and Treatment of COVID-19
Source: Rev Bras Enferm. 2026 Nov 6;79:e20250349. doi: 10.1590/0034-7167-2025-0349 (PMC13743049; doi:10.1590/0034-7167-2025-0349)
Supplement: Translation

# Translation: Itinerários Terapêuticos da População em Situação de Rua Para o Diagnóstico e Tratamento da COVID-19

**DOI:** 10.1590/0034-7167-2025-0349pt

**Authors:** Olívia Paulino Pinto, Ana Patrícia Pereira Morais, Mardenia Gomes Vasconcelos Pitombeira, Ana Paula Cavalcante Ramalho Brilhante, Maria Rocineide Ferreira da Silva

**Affiliations:** I Universidade Estadual do Ceará. Fortaleza, Ceará, Brasil; II Centro Universitário Christus. Fortaleza, Ceará, Brasil

**Keywords:** Itinerário Terapêutico, COVID-19, Política de Saúde, Pessoas Mal Alojadas, Avaliação em Saúde.

## Abstract

**Objetivo:**

compreender os itinerários terapêuticos de pessoas em situação de rua em busca do diagnóstico e tratamento de COVID-19.

**Métodos:**

estudo qualitativo e retrospectivo, realizado em Fortaleza, Ceará, por meio de entrevista semiestruturada. Os dados foram organizados no IRAMUTEQ^®^ e analisados pela matriz de análise “itinerários terapêuticos”, orientada pela análise holística de itinerários terapêuticos de Leny Trad e pelo sistema de atenção à saúde de Kleinman.

**Resultados:**

a descoberta do diagnóstico foi guiada pela descoberta em espaços formais de saúde e redes informais de apoio. A Atenção Primária à Saúde não foi acessada, percebendo-se que esse nível de atenção foi menos atuante, priorizando um cuidado mais biomédico. Relataram o uso de álcool e de outras drogas para o alívio dos sintomas da COVID-19.

**Considerações finais:**

os itinerários demonstraram a deficiência no acesso dos sujeitos, na resolução e na integralidade entre os serviços.

## INTRODUÇÃO

A situação de rua é caracterizada como uma crise global de direitos humanos, marcada pela negação da identidade social, pela ausência de moradia, pela criminalização e pelos direitos socioeconômicos extintos. A Política Nacional para a População em Situação de Rua define as pessoas em situação de rua (PSRs) como um grupo populacional heterogêneo que tem, em comum, o enfrentamento da pobreza extrema, vínculos familiares rompidos ou frágeis e o uso de espaços públicos como local de moradia e/ou sustento^(1-3)^.

As PSRs estão sujeitas a uma vulnerabilidade social e programática, com dificuldades persistentes e reconhecidas de acesso ao Sistema Único de Saúde (SUS) e aos apoios sociais. Isso resulta em elevada suscetibilidade a infecções devido às precárias condições que encontram nas ruas^(4)^. Diante disso, a chegada da doença COVID-19 (*Coronavirus Disease* 2019) intensificou a vulnerabilidade das PSRs, pois a infecção causada por esse vírus revelou-se altamente contagiosa e mortal, principalmente em indivíduos suscetíveis^(4,5)^.

Logo, apesar de atingir qualquer indivíduo, as questões macrossociais têm relação com o seu agravamento, sendo inegável que os maiores afetados são os grupos vulnerabilizados^(6)^. A vulnerabilidade socioeconômica aumentou de modo significativo depois da pandemia, contribuindo com a ampliação no contingente de PSRs^(7)^: cerca de 221 mil PSRs, o que já representava um aumento de 140% desde setembro de 2012^(8)^. Em relação à COVID-19, foram registrados no Brasil, até 2026, 39.318.227 casos e 716.626 óbitos pela doença. Esses números demonstram a extensão da pandemia desde sua deflagração em 2020, apresentando impacto profundo na saúde dos brasileiros^(9,10)^.

O contexto evoca reflexões sobre o cuidado da PSR durante a pandemia. Para tanto, a investigação adotou a teoria e a prática do itinerário terapêutico (IT), que têm sido estudadas para compreender a forma como certos grupos sociais fazem suas trajetórias até a assistência necessária^(11)^. O estudo é relevante porque possibilita uma melhoria nos fluxos de cuidado nas diferentes redes de atenção à saúde, garantindo o direito social e permitindo que os usuários expressem sua trajetória dentro do sistema. O estudo também contribui para a produção de conhecimento para a saúde coletiva nas áreas de ciências sociais, planejamento, gestão e avaliação de políticas públicas.

## OBJETIVO

Compreender os ITs de PSRs em busca do diagnóstico e tratamento da COVID-19.

## MÉTODOS

### Aspectos éticos

O estudo seguiu as regulamentações da Resolução nº 466/2012 do Conselho Nacional de Saúde, que dispõe sobre a pesquisa com seres humanos, e da Resolução nº 510/2016, que determina diretrizes específicas para as ciências humanas e sociais. Foi aprovado pelo Comitê de Ética em Pesquisa da Universidade Estadual do Ceará. Para o acesso aos participantes, foi solicitada autorização da Secretaria Municipal dos Direitos Humanos e Desenvolvimento Social para a realização da pesquisa no Centro de Referência Especializado para População em Situação de Rua (Centro Pop), e o consentimento para realização das entrevistas no espaço Casa da Sopa foi dado pelos voluntários responsáveis. Na abordagem às PSRs, foi apresentado o Termo de Consentimento Livre e Esclarecido (TCLE). Para garantir o sigilo e anonimato dos participantes, foram utilizados códigos numéricos nos roteiros de coleta de dados.

### Referencial teórico-metodológico

A matriz de análise utilizada foi IT. Entre os referenciais teóricos dos IT, foi escolhida para interpretação do material a análise holística de ITs proposta por Leny Trad^(12)^. O modelo apresenta quatro eixos interdependentes de análise: dimensão simbólica - responsável por sentidos, saúde-doença-cuidados; dimensão contextual - representa as condições de vida, o capital econômico, social e cultural, e a rede de suporte social; avaliação da experiência - aspectos organizacionais, relacionais, de eficácia e terapêuticos; racionalidade e práticas terapêuticas - relativas às práticas domésticas e outras arenas assistenciais terapêuticas^(12)^. Além dessa, foram considerados para análise os modelos explicativos da Kleinman^(13)^. Eles são definidos como concepções sobre a doença e formas de tratamento empregadas por todos aqueles engajados em um processo de saúde-doença.

### Tipo de estudo

Tratou-se de uma pesquisa com abordagem qualitativa e retrospectiva. A pesquisa atendeu aos itens recomendados pelo *Standards for Reporting Qualitative Research*.

### Cenário do estudo

A pesquisa foi realizada em Fortaleza, Ceará, cidade localizada no Nordeste do Brasil que, segundo o censo de 2025, possui 2,57 milhões de habitantes e conta com cerca de 10.037 PSRs. A pesquisa teve como cenário dois locais: o Centro Pop e o Grupo Espírita Casa da Sopa. O Centro Pop oferece acesso à documentação oficial, Cadastro Único Para Programas Sociais, higiene pessoal, atividades socioeducativas e artísticas, além de atendimento especializado, inclusive de saúde. A Casa da Sopa é uma instituição de referência, do tipo Organização da Sociedade Civil, que atua no desenvolvimento do cuidado social, promovendo a defesa dos direitos da PSR e oferecendo atividades no campo da assistência social^(14)^.

### Fonte de dados

Foram incluídas pessoas vivendo em situação de rua, no período da pandemia de 2020-2023, com diagnóstico de COVID-19, sem a necessidade de apresentação de teste comprobatório e maiores que 18 anos. Foram excluídas aquelas que não tinham condições de se comunicar, seja por apresentarem transtornos cognitivos que inviabilizassem a comunicação durante a coleta, seja por não falarem português, seja por estarem sob o efeito de álcool ou outras substâncias psicoativas. A seleção foi realizada por meio da técnica de coleta amostragem em bola de neve, que é uma forma de amostra que utiliza uma cadeia de referência, do tipo não probabilística^(15)^. O critério utilizado para finalizar a busca por participantes foi a saturação teórica dos depoimentos. Não houve recusa dos participantes abordados.

### Coleta e organização dos dados

As entrevistas foram conduzidas por meio de um roteiro semiestruturado entre setembro e outubro de 2023 e entre janeiro e março de 2024. Elas foram segmentadas em duas partes: a primeira refere-se aos dados sociodemográficos; e a segunda refere-se ao roteiro, que contém as seguintes perguntas disparadoras: você pode me falar como foi a busca para descobrir e saber da sua doença? Você pode me falar sobre o caminho que percorreu para fazer o tratamento? Por que escolheu esse caminho? Essas entrevistas foram realizadas pela pesquisadora principal em seu período de mestrado, de forma individual e de acordo com a disponibilidade do participante. Duraram entre 30 e 40 minutos, foram gravadas após consentimento verbal e assinatura do TCLE e foram transcritas na íntegra pela pesquisadora. A organização dos dados foi realizada por meio do programa informático *Interface de R pour les Analyses Multidimensionnelles de Textes et de Questionnaires* (IRAMUTEQ^®^), cuja utilização é gratuita. A ferramenta permitiu a identificação da raiz semântica das palavras e a extração das classes enunciadas, permitindo a consolidação da Classificação Hierárquica Descendente (CHD).

### Análise dos dados

A análise dos caminhos apresentados foi transcrita e identificada, inicialmente, por ordem de abordagem dos participantes. Após essa etapa, o material foi lido na íntegra e colocado para organização na ferramenta escolhida. A leitura foi necessária para a constituição do *corpus*, formulação e reformulação de pressupostos. A sustentação teórica foi possibilitada pelos ITs. Por meio dos ITs, é possível compreender que os sujeitos baseiam suas escolhas de acordo com suas necessidades, crenças, concepções de vida, nível de instrução e estigmas, ou seja, desenham e definem seus caminhos no sistema de saúde, independentemente do preestabelecido^(16)^.

As falas dos seis participantes foram compiladas em um *corpus* e processadas com o *software* IRAMUTEQ^®^. A CHD reconheceu seis classes textuais, 109 segmentos textuais, 3.701 ocorrências e 960 formas ativas, com apresentação média de ≥ 3:147. Destaca-se que o *corpus* textual deteve um aproveitamento de 80,73%, com vocábulos apresentando relevância estatística de associação significativa (p<0,0001). Foram geradas quatro categorias: Narrando o aparecimento da doença e os sentimentos envolvidos em sua descoberta; Rede de apoio social às pessoas em situação de rua na pandemia; Uso de álcool e outras drogas pelas pessoas em situação de rua no período da pandemia; Considerações sobre a Atenção Primária à Saúde no cuidado às pessoas em situação de rua com COVID-19.

## RESULTADOS

Foram entrevistados seis indivíduos, sendo uma entrevista coletada no Centro Pop e cinco no Grupo Espírito Casa da Sopa. A seguir, seguem os sujeitos e a descrição dos caminhos que percorreram: P1 - O participante era um homem de 57 anos, solteiro, católico, com um filho, natural de Fortaleza, autodeclarado branco, cisgênero, heterossexual e com ensino médio completo. Encontrava-se em situação de rua há 22 anos e fazia uso do Aluguel Social. O diagnóstico de COVID-19 foi identificado em 2021, por meio de testagem sistemática realizada pela Comunidade Católica Shalom, durante a entrega de alimentos em praça. Até a confirmação de seu diagnóstico, o participante havia realizado dez testes com resultado negativo. Após saber do diagnóstico, a primeira ação foi comprar bebidas alcoólicas e aderir ao isolamento. No relato, o participante afirmou estar assintomático, embora não tivesse certeza devido ao consumo de bebida alcoólica. Ele acreditava que o álcool poderia ter efeitos nocivos contra o coronavírus. Durante esse período, não houve busca por serviços de saúde e, após permanecer em isolamento domiciliar por duas semanas, retornou ao espaço público anteriormente ocupado (Figura 1).

**Figura 1:** Itinerário terapêutico de P1. Fortaleza, Ceará, Brasil, 2026

P2 - O participante era um homem de 35 anos, católico, divorciado, com três filhos, natural de Fortaleza, autodeclarado pardo, cisgênero, heterossexual e com ensino fundamental incompleto, que trabalhava como catador de material reciclável. Encontrava-se em situação de rua há mais de quatro anos e fazia uso de Aluguel Social. A origem da infecção foi atribuída pelo participante à internação hospitalar, referindo ter descoberto a doença logo após sua alta. A suspeita da doença ocorreu a partir do surgimento do desconforto respiratório e da diminuição do olfato e paladar. Diante dos sintomas, foi para a Unidade de Pronto Atendimento (UPA) 24 horas, fez o teste e os profissionais orientaram o isolamento. O participante tinha a crença de que o uso de drogas poderia ajudá-lo em sua cura. Assim, ao chegar em casa, fez uso de cocaína para aliviar o desconforto respiratório. Segundo o participante, houve melhora seguida de piora. Quando o desconforto aumentou, retornou para a UPA 24 horas, mas os profissionais mantiveram a intervenção anterior; após isso, retornou três vezes para a unidade. Relatou que Deus e o cigarro o ajudaram a passar por esse momento (Figura 2).

**Figura 2:** Itinerário terapêutico de P2. Fortaleza, Ceará, Brasil, 2026

P3- O participante era um homem de 39 anos, evangélico, solteiro, sem filhos, natural de Fortaleza, autodeclarado pardo, cisgênero e heterossexual, com ensino fundamental incompleto. Esteve em situação de rua por sete meses durante a pandemia, e encontrava-se nessa situação há um ano. A infecção por COVID-19 ocorreu em 2020, manifestando-se com febre e desconforto respiratório. À época, o indivíduo encontrava-se em situação de rua e, durante esse período, também vivenciou privação de liberdade. O diagnóstico foi confirmado através de *Reverse Transcription Polymerase Chain Reaction*, no momento em que aconteceu a admissão no sistema prisional. Após ser diagnosticado, foi encaminhado para uma unidade terciária; dessa, foi transferido para outro hospital, onde fez o isolamento e tratamento. Concluído o tratamento, retornou para a unidade de origem. Nesse período, o principal apoio que recebeu foi atribuído à equipe de enfermagem (Figura 3).

**Figura 3:** Itinerário terapêutico de P3. Fortaleza, Ceará, Brasil, 2026

P4 - O participante era um homem de 38 anos, evangélico, em união estável, com três filhos, natural de Fortaleza, autodeclarado pardo, cisgênero, heterossexual, ensino médio completo e desempregado. Encontrava-se em situação de rua há dez anos. Seu IT teve início quando sentiu febre e dores no corpo. Ao notar os sintomas, pediu ajuda à sua irmã, que o acompanhou até a UPA 24 horas. O participante contou que possuía um vínculo especial com essa unidade, recorrendo frequentemente a esse serviço, em que os profissionais o reconheciam. Na UPA 24 horas, foi confirmado o diagnóstico de COVID-19, sendo indicada a internação hospitalar. O participante foi transferido para uma unidade terciária e, durante esse período, apresentou desconforto respiratório e febre. Após 20 dias, teve alta e retornou para a rua (Figura 4).

**Figura 4:** Itinerário terapêutico de P4. Fortaleza, Ceará, Brasil, 2026

P5 - O participante era um homem de 27 anos, sem religião, casado, com quatro filhos, natural de Fortaleza, pardo, transgênero e bissexual. Estava em situação de rua há nove anos e, à época, era beneficiário do Aluguel Social. A infecção por COVID-19 ocorreu em 2020. Encontrava-se em situação de rua quando iniciou um quadro de febre e desconforto respiratório, e apesar de suspeitar ser COVID-19, não buscou assistência. O diagnóstico foi confirmado por meio de testagem, após admissão no sistema prisional. Após ser diagnosticado, foi encaminhado para isolamento, descrevendo a experiência como precária, incluindo o uso de gás lacrimogêneo e água dentro da cela por agentes penitenciários. A comida ofertada era estragada. Durante esse período, não teve apoio, e após cumprir o período de isolamento, voltou para a unidade de origem. Relatou receber apoio de Deus (Figura 5).

**Figura 5:** Itinerário terapêutico de P5. Fortaleza, Ceará, Brasil, 2026

P6 - O participante era um homem de 54 anos, evangélico, viúvo, sem filhos, natural de Fortaleza, autodeclarado negro, cisgênero, heterossexual e não era alfabetizado. Encontrava-se em situação de rua havia seis anos. Foi vítima de agressão na rua, com fratura na perna por objeto contundente, sendo necessária intervenção cirúrgica. Pouco tempo depois do procedimento, deu início aos sintomas, como desconforto respiratório, tontura e indisposição. Após diagnóstico positivo, foi transferido para uma área de isolamento, e além dos sintomas da doença, relatou que começou a ouvir vozes e ver vultos escondidos no quarto. De acordo com o participante, seu maior apoio foi o pastor, que o ajudou após a alta hospitalar. Ele finalizou o tratamento na Casa de Recuperação da Igreja Batista do Sétimo Dia, um espaço que o auxiliou com os sintomas pós-COVID e a recuperação da queda. Revelou em sua fala que: “Eu senti desespero e vontade de me matar [...] na verdade, eu sempre me ajoelho, sabe? Eu peço muita força a Deus” (Figura 6).

**Figura 6:** Itinerário terapêutico de P6. Fortaleza, Ceará, Brasil, 2026

## DISCUSSÃO

### Narrando o aparecimento da doença e os sentimentos envolvidos em sua descoberta

Os ITs realizados pelos participantes desta pesquisa não foram guiados somente pelas buscas, mas pela descoberta do diagnóstico em espaços formais de saúde ou pelas redes informais de apoio. Por isso, destaca-se a importância do acesso dessa população à rede formal de saúde, pois entende-se que os indivíduos que não foram alcançados pelas intervenções dos equipamentos de saúde ficaram suscetíveis ao agravamento do seu quadro e à transmissão da doença.

Foi identificado nas falas que o desconforto respiratório se tornou, pela percepção dessa população, um sintoma identificador da doença. Entretanto, a maioria das pessoas apresenta sintomas leves (40%) ou moderados (40%), e apenas 15% desenvolvem sintomas que requerem suporte de oxigênio^(17)^. Assim, a procura dos pacientes que tiveram casos leves pode ter sido prejudicada pela falta de conhecimento dos sinais e sintomas. A falta de conhecimento acerca dos sinais e sintomas, a demora do usuário para acessar um serviço de saúde, a falta de serviços de referência e as falhas estruturais e operacionais da Rede de Atenção Básica dificultam o percurso para acesso ao diagnóstico^(18)^.

Durante a pandemia, diversos fatores contribuíram para a baixa incidência de positividade nessa população. É fato que as dificuldades de acesso podem ter implicado casos não diagnosticados, e outros fatores, como a falta de documentação e a inexistência de um marcador, também influenciaram os óbitos da PSR nos censos. Não houve registro do número de casos e óbitos nesses indivíduos em Fortaleza, dificultando as ações da Vigilância Epidemiológica^(19)^.

As reflexões geradas pela construção dos ITs revelam as lacunas no cuidado e na operacionalização do sistema de saúde. Assim, precisa operar para além de uma visão funcionalista, levando em consideração as demandas sociais e adequando a oferta de práticas e serviços em saúde^(16)^.

Os sentimentos e as percepções dos usuários tiveram papel importante na recuperação e no comportamento diante do adoecimento. Assim como no relato do participante 6, a doença foi vista como desesperadora, mas a partir de uma ótica de esperança e superação. Devido ao estado de pânico disseminado em parte pela mídia, esperava-se que os sentimentos relatados fossem negativos. Porém, a maioria dos entrevistados mostrou um enfrentamento positivo, geralmente motivado por um aspecto religioso. Essa crença desempenha principalmente o papel de enfrentar ameaças e renovar as energias na luta pela sobrevivência^(20)^.

### Rede de apoio social às pessoas em situação de rua na pandemia

A rede de apoio social é definida como o conjunto de pessoas com as quais se estabelecem relacionamentos significativos, percebidos e/ou recebidos pelos indivíduos. Esse conceito é de extrema importância para a compreensão da produção do cuidado de saúde, pois, se a rede é integrada e forte, tem a capacidade de proporcionar resiliência no atravessamento de crises^(21)^. Para entender as escolhas terapêuticas realizadas, é necessário identificar essas redes. Elas podem ser compostas de contatos íntimos, mas também de arenas de cuidados formais e informais, pois é a partir dessas redes que o indivíduo vivencia sua doença e se mobiliza para o cuidado^(22)^.

Analisou-se aqui a dimensão de contexto, que consiste em um plano que reúne informações relativas ao território de vida, considerando a infraestrutura básica e sociosanitária do local. Cabe dimensionar a extensão e as especificidades dos recursos assistenciais presentes, considerando modalidades formais e informais de serviço^(12)^. A pandemia exigiu adaptação e reorganização assistencial em todos os níveis de complexidade para o atendimento eficiente das pessoas diagnosticadas com COVID-19, sendo realizada a ampliação de leitos hospitalares, a contratação de profissionais, o fornecimento de equipamentos de proteção individual, locais para isolamento, a elaboração de novos fluxos assistenciais e protocolos, capacitações, entre outras ações necessárias^(23)^.

Para isso, foi realizada a síntese das redes de apoio formais e informais que surgiram nas falas das PSRs acerca dos equipamentos utilizados para a busca do diagnóstico e o tratamento da doença. Entre os citados, foi observado que a arena profissional teve maior destaque entre os equipamentos de busca das pessoas entrevistadas. Locais como as UPAs 24 horas e os hospitais foram colocados como os mais acessados.

Observou-se a influência da arena *folk* e informal para o apoio no momento de adoecimento, com destaque para amigos, familiares e organizações religiosas. Os atores sociais buscaram cuidado e tratamento por meio de diversas articulações na rede social, constituindo ITs singulares que apresentaram ações baseadas em dúvidas, vacilações e na utilização de múltiplos recursos. Por fim, esses setores não devem ser pensados de forma individualizada. Kleinman destaca a necessidade de integração entre os setores para que o cuidado seja realizado de maneira integral^(13)^.

### Considerações sobre a Atenção Primária à Saúde no cuidado às pessoas em situação de rua com COVID-19

No mapeamento dos equipamentos que compõem a arena profissional, a maioria dos participantes apresentou níveis de atenção com complexidade secundária/terciária e instituições não governamentais. No entanto, se, por um lado, existe a efetivação dos direitos das PSRs por esses atores, por outro, discutimos as lacunas deixadas pela Atenção Primária à Saúde (APS) e pelos serviços de referência^(24)^. O potencial apresentado pela APS no enfrentamento de crises é decisivo, servindo principalmente na orientação comunitária e no reconhecimento das necessidades dentro dos territórios, contribuindo por meio da análise epidemiológica e possuindo papel essencial na proteção da população vulnerável, como as PSRs^(24)^.

Entretanto, os documentos publicados pelo Ministério da Saúde (MS) reforçaram o manejo clínico da COVID-19, revelando protocolos rígidos de testagem apenas em casos sintomáticos, classificando os casos suspeitos como síndrome gripal inespecífica. Por conseguinte, a APS não recebeu estrutura e/ou orientação em documentos do MS para estabelecer a busca ativa de casos suspeitos ou coordenar as testagens, induzindo a concentração de testagem em níveis hospitalares^(24)^. Em relação ao plano de contingência de Fortaleza, o texto destaca o atendimento e o acompanhamento regular dos pacientes suspeitos ou confirmados com a doença. Ou seja, o foco é depositado em uma dimensão biomédica de cuidado^(25)^.

Direcionando as ações ao público de estudo, em 2020, a Secretaria de Saúde do Estado do Ceará lançou uma nota técnica com recomendações para a PSR. O objetivo foi orientar medidas de prevenção e proteção desse público nos diferentes níveis de atenção. No caso de os municípios não possuírem Consultório na Rua, equipamento que compõe a APS, desenvolvendo ações integrais para PSRs, a APS ficaria responsável pela oferta de cuidado especializado a essa população^(26)^. No entanto, as falas revelam que tiveram acesso a outras instâncias, expondo que as medidas sugeridas pelas recomendações tiveram pouco impacto ou não foram realizadas dentro do município^(27)^.

Em crises sanitárias, a APS possui a capacidade de assumir uma ação protagonista, devido à sua vinculação ao território, à sua resolutividade no trato de epidemias e ao cuidado integral. Isso a torna de grande importância no enfrentamento do adoecimento coletivo por uma afecção desconhecida, agravada por um cenário de desproteção social, de insegurança alimentar, de desemprego e de violências. Entretanto, observou-se o esvaziamento de ações no enfrentamento da crise, resultando na imposição de um não lugar para esse nível de atenção, apesar de seu grande potencial de atuação dentro da rede de saúde^(16)^.

Os países localizados na América Latina, como o Brasil, enfrentavam problemas de cunho econômico, político e social. A gestão pública e o tempo de resposta foram fundamentais na condução dessa pandemia. Mesmo países que possuíam os ativos econômicos foram capazes de evitar o adoecimento e a morte de sua população como consequências das escolhas feitas por seus gestores locais e de saúde^(28)^.

### Uso de álcool e outras drogas pelas pessoas em situação de rua no período da pandemia

A situação de rua está associada às vulnerabilidades de saúde, sociais e legais, exacerbadas pelo abuso de substâncias psicoativas, que assume, nesse contexto, a função de auxiliar na socialização e minimizar a fome e o frio^(29)^. Essa arena contextual, prevista nas dimensões, conta com desigualdades, acesso dificultado a serviços básicos e políticas públicas insuficientes, dificultando o rompimento dessa vulnerabilidade^(12)^.

O consumo de substâncias por essa população possui múltiplos significados e símbolos, o que ressalta a necessidade de uma compreensão aprofundada para que políticas públicas sejam formuladas de maneira contextualizada e eficaz^(30)^.

Em alguns casos, o uso de substâncias é percebido pelos próprios indivíduos como terapêutico, uma estratégia de alívio ou cura de dores e/ou sofrimentos psíquicos. É nesse contexto que se visualiza a dimensão simbólica, por apresentar representações e significados relativos ao processo de saúde-doença. Nesse caso, a percepção positiva da droga afeta diretamente a saúde e o processo de adoecimento desses indivíduos. Em outra esfera de investigação, o percurso terapêutico transita pela análise das racionalidades subjacentes e das práticas terapêuticas das diferentes arenas de cuidado. Na pesquisa de campo, observou-se o *déficit* no autocuidado e o apoio social, condição presente no uso de substâncias psicoativas como terapêutica para a doença^(12)^.

Faz-se necessário abordar o uso de substâncias nos ITs descritos, pois o isolamento social e a nova dinâmica vivenciada criaram ambiente propício para o uso de substâncias, afetando, em especial, aqueles indivíduos que faziam uso de álcool e outras drogas anteriormente^(31)^. A fragilização gerada pelo isolamento e desconhecimento sobre a doença corrobora o estudo de Jr. e Ximenes^(32)^, que evidencia que a utilização da droga contribui para a mediação do sofrimento e do incômodo vivenciados por estar adoecido, proporcionando sensação de prazer imediato e alívio, além de familiaridade quando há uso prolongado de substâncias psicoativas.

Os significados trazidos pelos participantes apresentam uma diversidade de contextos, simbolismos e racionalidades. Porém, ressaltam-se nesses ITs lacunas deixadas pelos níveis de atenção. Ao comparar o contexto do Brasil com o América do Norte e Europa, observa-se que houve maior apoio e urgência em alocar recursos. A expansão dos serviços de redução de danos e tratamento, além da intersetorialidade entre serviços, possibilitou o aumento do acesso aos serviços de saúde mental para os indivíduos^(33)^.

### Limitações do estudo

O desafio de abordagem ao público foi a limitação apresentada, pois a maioria dos participantes não possuía conhecimento do próprio diagnóstico. Para uma avaliação mais qualificada da temática, seria necessária uma investigação em outras regiões e uma amostra maior.

### Contribuições para a área da enfermagem, saúde ou políticas públicas

No campo da saúde coletiva e da enfermagem, a utilização de ITs possibilita uma análise mais assertiva das necessidades das PSRs, pois é a partir do olhar do usuário que conseguimos criar planos de contingência capazes de atender às demandas de saúde em cenários de emergência. Nesta lógica, os ITs são um método de avaliação que possibilita a construção compartilhada das políticas.

## CONSIDERAÇÕES FINAIS

A construção desses trajetos evidenciou que a busca pelo diagnóstico é elaborada com base na gravidade do adoecimento e nos vínculos formados previamente à pandemia, seja com as unidades de saúde, com familiares ou com figuras religiosas. Os resultados destacaram a falta de estrutura, organização e redes articuladas para o acesso das PSRs, demonstrando fragilidades na resolutividade e na integralidade dos serviços. Considerando a trajetória descrita pelos usuários, ficou evidente a desarticulação da rede, que deixa o tratamento centralizado na assistência secundária e terciária, bem como em instituições não governamentais.

É importante qualificar o trabalho entre as redes para garantir uma melhor resposta em momentos de crise. A construção de IT tem a potencialidade de fomentar, potencializar e, certamente, melhorar as iniciativas de articulação e cooperação entre espaços e sujeitos da gestão ou da produção de saúde nos serviços e contextos diversos.

## Data Availability

Os dados da pesquisa estão disponíveis mediante solicitação.
